# Effects of dietary antimicrobial peptides on growth performance, antioxidant capacity, immune response and disease resistance of juvenile Nile tilapia (*Oreochromis niloticus*)

**DOI:** 10.3389/fphys.2026.1936199

**Published:** 2026-09-11

**Authors:** Wencong Zhang, Lei Wang, Jinliang Zhao, Biao Yun, Xueqiao Qian

**Affiliations:** 1 Department of Aquatic Germplasm and Genetic Breeding, Animal Husbandry and Fisheries Research Center of Guangdong Haid Group Co., Ltd., Guangzhou, China; 2 College of Fisheries and Life Science, Shanghai Ocean University, Shanghai, China

**Keywords:** antimicrobial peptide, antioxidant capacity, disease resistance, growth, immune, Nile Tilapia

## Abstract

**Introduction:**

The study was conducted to investigate effects of dietary antimicrobial peptide, tilapia-derived endogenous piscidin 4 (TP4), on growth performance, antioxidant capacity, immune response and disease resistance of juvenile Nile Tilapia (*Oreochromis niloticus*).

**Methods:**

Four diets with supplementation of 0 g/kg (Control), 2.0 g/kg, 4.0 g/kg, and 8.0 g/kg of TP4, respectively, were prepared for tilapia (initial weight 11.67 ± 0.21 g).

**Results:**

Results showed that specific growth rate of fish fed the diet with 2.0 g/kg TP4 was significantly higher, while feed conversion ratio was lower than those in the control (P < 0.05). Meanwhile, 2.0 g/kg TP4 supplementation markedly elevated crude protein contents and condition factor, as well as intestinal trypsin and lipase activities than the control (*P* < 0.05). In addition, 2.0 and 4.0 g/kg TP4 supplementation enhanced antioxidant capacity, evidenced by significantly increased catalase and peroxidase activities, as well as decreased malondialdehyde contents in liver (*P* < 0.05). Moreover, the expression of antioxidant genes *sod1*, *cat*, *nrf2* and *atf4* was higher significantly in 2.0 and 4.0 g/kg TP4 groups than in the control (*P* < 0.05). Also, 2.0 g/kg TP4 supplementation significantly reduced activities of glutamic-oxalacetic transaminase and glutamic-pyruvic transaminase and elevated the activity of alkaline phosphatase (*P* < 0.05). Relatedly, pro-inflammatory genes *il8* and *tnfα* were downregulated, anti-inflammatory genes *il10* and *arg1* were upregulated, and relative percent survival of fish after challenge with *Streptococcus agalactiae* was elevated in the 2.0 g/kg TP4 group than in the control, demonstrating that 2.0 g/kg TP4 may improve immune response and disease resistance. However, these favorable effects on antioxidant capacity and immunity disappeared at the 8.0 g/kg TP4 supplementation level.

**Discussion:**

In conclusion, 2-4 g/kg TP4 supplementation in the diet effectively enhanced antioxidant capacity, immune response and disease resistance, which may potentially improve the growth performance.

## 1 Introduction

The rapid development of intensive and high-density aquaculture has aggravated environmental stress in culture water (Pikelj et al., 2022), leading to frequent outbreaks of bacterial diseases in farmed fish (Zhang et al., 2022b). Antibiotics have long been the primary strategy for disease prevention and control in aquaculture (Wang et al., 2021b). However, their indiscriminate application readily induces the emergence of drug-resistant bacterial strains, accompanied by drug residues and disrupted aquatic microecology (Dong et al., 2018; Ma et al., 2022). These hazards severely hinder the environmentally sustainable development of aquaculture (Choi et al., 2022). Accordingly, developing safe, efficient, residue-free feed additives that barely trigger bacterial resistance has become a key research focus in aquatic nutrition and disease prevention.

Antimicrobial peptides (AMPs) are small bioactive polypeptides secreted by the innate immune system of organisms (Li et al., 2019b). With broad-spectrum bacteriostatic activity, low toxicity, zero residue and low risk of inducing bacterial resistance, AMPs are promising alternatives to antibiotics (Zhao et al., 2021). Recent research has proven that dietary AMP supplementation exerts beneficial effects on growth performance, antioxidant capacity and immune function of mammalian and poultry species (Xiao et al., 2013; Shi et al., 2017; Cheng et al., 2022; Shen et al., 2022; Gao et al., 2023; Horng et al., 2025). Likewise, studies on orange-spotted grouper (*Epinephelus coioides*), common carp (*Cyprinus carpio*) and large yellow croaker (*Larimichthys crocea*) have verified that AMPs promote nutrient digestion utilization efficiency and further growth of aquatic animals (Zhai et al., 2017; Dong et al., 2019; An et al., 2023). Meanwhile, trials conducted on orange-spotted grouper and largemouth bass (*Micropterus salmoides*) revealed that dietary AMPs markedly enhance antioxidant enzyme activities and alleviate oxidative damage triggered by aquaculture stressors (Su et al., 2019; Li et al., 2020). In addition, investigations on Koi (*Cyprinus carpio koi*), grass carp (*Ctenopharyngodon idellus*) and tsinling lenok trout (*Brachymystax lenok tsinlingensis*) reported that AMPs effectively modulate the expression of immune-related factors and optimize the non-specific immune response, reducing post-pathogen infection mortality and improving anti-infection capacity (Lin et al., 2013; Liu et al., 2020; Ma et al., 2024). Most existing studies focus on exogenous AMPs and adopt low-temperature pelleting or post-spray coating to avoid thermal degradation, whereas systematic evaluations of endogenous AMPs under conventional extrusion feed processing remain scarce.

Nile Tilapia (*Oreochromis niloticus*) is a dominant freshwater aquaculture species in China (Chen et al., 2019; Xia et al., 2020). Massive outbreaks of *Streptococcus agalactiae* frequently occur during high-temperature seasons, causing severe economic losses to the aquaculture industry (Liang et al., 2020; Zhang et al., 2022d). Compared with AMPs from alternative origins, fish-derived peptides exhibit great potential as antibiotic alternatives for mitigating bacterial infections in cultured fish (Pradhan et al., 2017; Chrom et al., 2019). In this study, the endogenous piscidin 4 (TP4) isolated from tilapia exhibits biocompatibility, rendering it less vulnerable to proteolytic degradation (Peng et al., 2012; Fei et al., 2025). Furthermore, TP4 exhibits moderate thermostability that maintains bactericidal activity upon short-term exposure to high-temperature, making it promising nutritional regulators in the extruded feed to simultaneously improve tilapia growth and health (Pan et al., 2017; Hao et al., 2021; Tai et al., 2021). Its thermostable mechanism may originate from a high proportion of cationic arginine residues within the peptide sequence, which strengthen intramolecular electrostatic interactions and stabilize amphipathic α-helical conformation against heat-induced unfolding (Bai et al., 2024). The stability assays *in vitro* verified TP4 retains full antibacterial and immunomodulatory activity after sustained exposure to 55~65°C, matching the drying temperature adopted during extrusion processing (Zhang et al., 2025).

Earlier studies on AMP in tilapia merely verified antibacterial activity *in vitro* (Hazam et al., 2023) or detected partial serum biochemical indicators (Chen et al., 2016). Few published work has simultaneously quantified whole-body nutrient deposition, intestinal digestive enzyme, hepatic antioxidant enzyme, antioxidant/immune gene transcription profiles and post-*S. agalactiae* challenge survival to form a complete physiological response chain for application of AMP in tilapia. Meanwhile, existing studies on AMP in tilapia have exclusively adopted pelleted feeds, which is inconsistent with the prevailing industrial application of extruded aquafeeds. Therefore, taking advantage of the moderate thermostability of TP4 and the limited research on AMP in tilapia, we investigated the effects of TP4 on growth performance, antioxidant status, immune response and disease resistance of juvenile Nile Tilapia when incorporated into extruded feeds.

## 2 Materials and methods

### 2.1 Feed production and storage

Four isonitrogenous (33%) and isolipidic (9.5%) diets with supplementation of 0 g/kg (Control), 2.0 g/kg (AMP-2), 4.0 g/kg (AMP-4), and 8.0 g/kg (AMP-8) of AMP, i.e., TP4, were prepared for juvenile tilapia, respectively. Microcrystalline cellulose served as a weight-for-weight replacement for AMP in all experimental diets. The measured concentrations of AMPs in four diets were 0.01 g/kg, 1.93 g/kg, 3.89 g/kg and 7.78 g/kg, respectively. The AMP, which is derived from Nile Tilapia and designated tilapia TP4, was obtained from the Animal and Aquatic Research Center of Guangdong Haid Group Co., Ltd. The feed formula is shown in **Table 1**. According to the feed formula, ingredients were thoroughly mixed, and passed through an 80-mesh sieve, and then processed into feed pellets via the extrusion. Afterward, all diets were dehydrated in a drying oven at 55°C for 11 h. Finally, diets were put into vacuum plastic bags and placed in a dry and well-ventilated storage room for later use.

**Table 1:** Ingredients and proximate composition of trial diets (g/kg dry matter).

Ingredients	Antimicrobial peptides supplementation level (g/kg)			
	Control	AMP-2	AMP-4	AMP-8
Fish meal^a^	50.0	50.0	50.0	50.0
Soybean meal^a^	320.0	320.0	320.0	320.0
Rapeseed meal^a^	100.0	100.0	100.0	100.0
Peanut meal^a^	170.0	170.0	170.0	170.0
Corn starch	245.0	245.0	245.0	245.0
Soybean oil	50.0	50.0	50.0	50.0
Soybean lecithin	10.0	10.0	10.0	10.0
Ca(H_2_PO_4_)_2_	20.0	20.0	20.0	20.0
Vitamin premix^b^	10.0	10.0	10.0	10.0
Mineral premix^c^	10.0	10.0	10.0	10.0
Sodium chloride	2.0	2.0	2.0	2.0
Choline chloride	4.0	4.0	4.0	4.0
Ethoxyquin	0.5	0.5	0.5	0.5
Calcium propionate acid	0.5	0.5	0.5	0.5
Microcrystalline cellulose	8.0	6.0	4.0	0.0
Antimicrobial peptide	0.0	2.0	4.0	8.0
Proximate composition (%)				
Crude protein	32.9	33.1	33.4	33.3
Crude lipid	9.7	9.6	9.8	9.8
Crude ash	10.2	9.9	10.3	10.5

^a^ Commercially available from Guangdong Haid Group Co., Ltd. (Guangzhou, China); elementary composition (dry matter): red fish meal: crude protein 64.1%, crude lipid 12.2%; soybean meal: crude protein 46.3%, crude lipid 1.0%; rapeseed meal: crude protein 36.4%, crude lipid 1.2%; Peanut meal: crude protein 52.1%, crude lipid 6.4%.

^b^ Commercially available from Guangdong Haid Group Co., Ltd. (Guangzhou, China); composition of vitamin premix (mg/kg diet): vitamin B_1_, 25; vitamin B_6_, 20; vitamin B_12_, 10; vitamin K_3_, 10; inositol, 800; folic acid, 20; pyridoxine-HCl, 20; niacin acid, 200; retinyl acetate, 32;α-tocopherol, 240; ascorbic acid 2000; riboflavin, 45; biotin (2%), 60; cholecalciferol, 5; thiamin, 25; microcrystalline cellulose, 6470.

^c^ Commercially available from Guangdong Haid Group Co., Ltd. (Guangzhou, China); composition of mineral premix (mg/kg diet): MnSO_4_·H_2_O, 45; Na_2_SeO_3_ (1%), 20; MgSO_4_·7H_2_O, 1200; CuSO_4_·5H_2_O, 10; Ca(IO_3_)_2_ (1%), 60; CoCl_2_·6H_2_O (1%), 50; FeSO_4_·H_2_O, 80; ZnSO_4_·H_2_O, 5; Zeolite, 3485.

A total of 40 kg of each diet was prepared to meet the feed demand of the rearing trial.

### 2.2 Fish farming and sampling operation

Juvenile tilapia used in the trial were obtained from Bairong Aquatic Seed Co., Ltd. (Dingan, China). Experimental fish were cultured in the Aquatic Research and Development Base of Guangdong Haid Group Co., Ltd. (Guangzhou, China). All fish were temporarily raised for 15 days to adapt to the environment and were fed with the same commercial diet (9% crude lipid and 33% crude protein contents). Then, healthy fish with consistent specifications (initial weight 11.67 ± 0.21 g) were selected for the formal experiment. The experiment was divided into 4 groups, with six replicates in each group. A total of 24 fiberglass tanks (400 L) were used with 30 tilapia per tank. Fish were randomly distributed to each tank. The 60-day feeding trial was conducted in a flowing water culture system, and daily testing was performed to ensure that the water temperature was 28-32°C, the dissolved oxygen was ≥6.0mg/L, and the pH was 7.5-8.5. Meanwhile, 60% of the water per tank was changed twice daily. All fish were fed to apparent satiety twice daily (8:00 a.m. and 7:00 p.m.) and the dead fish were recorded.

After the feeding experiment, fish were starved for 24 h. Subsequently, the remaining fish per tank were counted and weighed. Prior to sampling, tilapia were anesthetized using 70 mg/L eugenol. The body length of fish was measured, and the weight of fish, viscera and liver was weighed. Then, the liver and intestine of every four fish were mixed into one cryovial, respectively. The cryovials containing samples were stored in a medical refrigerator at -80°C for subsequent detection. Four fish per tank were collected and kept frozen in a freezer at -20°C for body compositional analysis.

### 2.3 Indicators calculation

The calculation formulas were as follows:

Weight gain rate (WGR, %) = 100 × (w_2_ – w_1_)/w_1_.
Specific growth rate (SGR, % day ^-1^) = 100 × (Ln w_2_ – Ln w_1_)/60 days.
Feed conversion ratio (FCR) = 100 × D_in_/(w_2_ – w_1_).
Feed intake (FI) = 100 × D_in_/(60 × (w_2_ + w_1_)/2).
Survival rate (SR, %) = 100 × n_2_/n_1_.
Hepatosomatic index (HSI, %) = 100 × (liver weight/fish weight).
Viscerosomatic index (VSI, %) = 100 × (viscera weight/fish weight).
Condition factor (CF) = 100 × (final body weight/final body length^3^).
Relative percent survival (RPS, %) = 100 × (D_CON_ – D_AMP_)/D_CON_.

where w_2_ is the final weight of fish per tank, w_1_ is the initial weight of fish per tank, n_2_ is the final number of fish per tank, n_1_ is the initial number of fish per tank, D_in_ is the dry diet consumed per tank, D_CON_ is the number of dead fish in the control group after challenge, D_AMP_ is the number of dead fish in groups supplemented antimicrobial peptides after challenge.

### 2.4 Proximate composition analysis

Feed nutritional composition, including crude lipid, protein and ash, as well as whole-body composition of tilapia, including moisture, crude lipid and protein were analyzed according to the previous methods (AOAC, 1995). Briefly, samples were first dried to constant weight in an oven at 105 °C to determine moisture content. Subsequently, crude protein content was measured by the Kjeldahl method using a Kjeltec™ 8400 analyzer. After soaking in petroleum ether for 24 h, crude lipid content was determined via the Soxhlet extraction method with an automatic Soxhlet extractor. Final, the samples in crucibles were first ignited on an electric furnace until smoke ceased and then subjected to dry-ashing in a muffle furnace at 550 °C for 15 h to determine crude ash content. The instruments and methods used for the analysis of the above indicators are shown in **Table 2**.

**Table 2:** Diets and fish body nutritional composition analysis.

Nutritional composition	Instruments	Methods	Samples
Moisture	A drying oven	Drying for 48 h to constant weight	Fish body
Crude lipid	Soxtec TM 8000, FOSS	Soxhlet extracted method	Diets and fish body
Crude protein	Kjeltec TM 8400, FOSS	Kjeldahl method	Diets and fish body

### 2.5 Biochemical testing

Intestinal digestive enzymes including lipase (LPS), trypsin (TRY) and α-amylase (AMS), as well as hepatic biochemical parameters including total antioxidant capacity (TAOC), superoxide dismutase (SOD), peroxidase (POD), catalase (CAT), malondialdehyde (MDA), lysozyme (LZM), glutamic-pyruvic transaminase (GPT), glutamic-oxaloacetic transaminase (GOT), alkaline phosphatase (AKP) and total protein (TP) were analyzed by reagent test kits purchased from Nanjing Jiancheng Biotechnology Company (Nanjing, China). The pre-processing steps of the intestine referred to the previous study (Zhang et al., 2022c).

### 2.6 Liver total RNA extraction and real-time quantitative PCR

Frozen liver samples were ground into powder in liquid nitrogen and 80 mg was weighed, followed by adding 1000uL Trizol reagent (Takara, China) and mixing thoroughly in a homogenizer. The subsequent RNA extraction steps were based on Zhang et al. (2022c). Then, a Nano-Drop spectrophotometer (Thermo Fisher Scientific, USA) was employed to obtain the concentration of the total RNA and assess the purity of the extracted RNA by calculating the ratios of 260/230 and 260/280. Agarose gel electrophoresis was adopted to determine the integrity of RNA. Afterward, PrimeScript™ RT reagent Kit (Takara, China) was used to reverse-transcribe the isolated RNA into single-stranded DNA as the subsequent qRT-PCR template.

The qRT-PCR used a 20uL reaction system, including 5uL cDNA template, 8uL Taq Pro Universal SYBR qPCR Master Mix (Vazyme, China), 0.5uL forward primers, 0.5uL reverse primers and 6uL ddH_2_O. Subsequently, MA-6000 Real-Time Fluorescence Quantitative PCR Instrument (Yarui, China) was employed for qRT-PCR and the reaction procedure was the same as Zhang et al. (2022c). The qRT-PCR program was set as follows: one cycle of 95 °C for 2 min, followed by 38 cycles of 95 °C for 10 s, 56.5 °C for 10 s, and 72 °C for 20 s. For gene quantification, three technical replicates were performed for each biological replicate to minimize operational errors. The melting curve and standard curve analysis were carried out to verify the PCR product specificity and PCR amplification efficiency, respectively. The amplification efficiency of genes ranged from 0.94 to 1.06. *β-actin*, which is stably expressed in the liver of tilapia (Li et al., 2019a), was used as an internal reference gene. The primer sequences of all genes are listed in **Table 3**. The Ct values obtained from the reaction were calculated using the 2^-ΔΔCt^ formula (Livak and Schmittgen, 2001).

**Table 3:** Primers used for real-time quantitative PCR in the study.

Genes	Forward primers (5′-3′)	Reverse primers (5′-3′)	Gene bank No.
*β-actin*	CAGCATCATGAAGTGCGACG	TATTTACGCTCAGGTGGGGC	KJ126772.1
*keap1*	TGTTGGGAGGTTACGATGGC	ACATCCTTTTGGCAGGGCTC	XM_003447926.3
*sod1*	CGTTTTGAAGGGAACTGGCG	GCACCCGTTTGTGTTGTCTC	100701098
*cat*	TGGTCAATGCCGATGGTGAA	TAATCTGGGTTGGTGGCTGC	XM_019361816.2
*nrf2*	AGGTTACACAGAATGCCGGT	GGCTGTTCGGTGGACATCAT	XM_003447296.4
*atf4*	ACCTCTCCACCAAGCGAATG	GTCTTGGGAGAGGTGGAAGC	XM_003459356.5
*myd88*	AAGATTCCTCTCGTTGCGCT	GCAGGTTGGGTTTTTGGACA	100700534
*il8*	GTGAAGGCATGGGTGTGGAG	CCTGTCCTTTTCAGTGTGGC	100534479
*tnfα*	GTCCTCCTGGCATTCACACT	CTGCCATTCCACTGAGGTCTT	100697746
*il10*	GCGTCTTTCTTCAGCTCTGC	TCCTGTCTGAGCTTCTTGAGC	100694754
*arg1*	GCCAGAGGGAAGAAACCGAT	TGTTCGGTGATGTAGACGCC	100706482

*keap1*, kelch-like ECH-associated protein 1; *sod1*, superoxide dismutase 1; *cat*, catalase; *nrf2*, nuclear factor E2-related factor; *atf4*, activating transcription factor 4; *myd88*, myeloid differentiation factor 88; *il8*, interleukin 8; *tnfα*, tumor necrosis factor α; *il10*, interleukin 10; *arg1*, arginase 1.

### 2.7 Bacterial challenge

At the termination of the feeding trial, 60 fish were randomly selected from each of the four dietary treatment groups and distributed into three tanks (20 fish per tank), generating three biological replicates per group for the subsequent *S. agalactiae* challenge test. In addition, another 60 fish were randomly sampled from the four treatment groups and divided into three biological replicates (20 fish per replicate), which were intraperitoneally injected with phosphate buffered saline (PBS) to serve as the vehicle control. The strain of *S. agalactiae* used in this study was preserved at the Animal and Aquatic Research Center of Guangdong Haid Group Co., Ltd. Fish were challenged via intraperitoneal injection with a bacterial suspension at a concentration of 1.0×10^7^ CFU/mL (determined based on the pre-experiment shown in **Supplementary Table 1**), with an injection volume of 0.1 mL per fish and the total bacterial load per fish of 1.0×10^6^ CFU. Bacterial suspension was freshly prepared prior to the challenge test. Briefly, the preserved *S. agalactiae* strain was streaked and cultured on blood agar plates at 37 °C overnight. Single colonies were picked and inoculated into liquid broth for continuous shaking incubation. After incubation, the bacterial culture was centrifuged and washed twice with sterile PBS to remove the medium residue. The bacterial pellet was resuspended and serially diluted with sterile PBS, and the final bacterial concentration was quantified via plate counting. All experimental fish were maintained under identical water quality and rearing management conditions post-challenge, and mortality was recorded daily for a consecutive 7 days.

### 2.8 Statistical analysis

SPSS software (SPSS version 25.0) was used for data statistics. One-way ANOVA was sued and subsequently Tukey’s test was adopted to perform multiple comparisons. *P* < 0.05 was statistically significant. Prior to ANOVA, the normality of residuals was assessed using the Shapiro-Wilk test, and homogeneity of variance was evaluated by Levene’s test. Besides, orthogonal polynomial contrasts were applied to evaluate linear and quadratic dose-response trends of AMP supplementation. The challenge test was performed with three biological replicates (n = 3), whereas all other assays were conducted with six biological replicates (n = 6). For biochemical and transcriptional detection, laboratory analysts performed sample testing without knowing the grouping information of samples, which represents a single-blind protocol. Figures in this study were drawn using Graph Pad software (Graph Pad version 9.0).

## 3 Results

### 3.1 Survival, growth performance and feed efficiency

There was no significant difference in SR among the four groups (*P* > 0.05). As dietary AMP levels increased to 4.0 g/kg, FBW, SGR and WGR showed a significant trend of first increase and then decrease (*P* < 0.05), and was then significantly elevated again in the AMP-8 group (*P* < 0.05). These parameters in the AMP-2 group were significantly higher than in other groups. However, FCR and FI were first decrease and then increase as dietary AMP levels increased to 4.0 g/kg (*P* < 0.05). There was no significant difference in FCR between the control and AMP-8 group (*P* > 0.05), while FI was significantly higher in the AMP-8 group than in the control group (*P* < 0.05) (**Table 4**).

**Table 4:** The effects of dietary antimicrobial peptides piscidin 4 (TP4) on survival, growth and feed utilization of juvenile Nile Tilapia for 60-day feeding trial.

Parameters	AMP supplementation level				Polynomial contrasts		
	Control	AMP-2	AMP-4	AMP-8	P-ANOVA	P-Linear	P-Quadratic
IBW/g	11.61±0.04	11.62±0.03	11.64±0.03	11.59±0.04	0.759	0.534	0.425
FBW/g	109.75±0.25c	112.58±0.13a	109.36±0.09c	111.15±0.21b	0.000	0.100	0.592
SR/%	100.00±0.00	100.00±0.00	100.00±0.00	99.45±0.56	0.413	0.144	0.430
WGR/g	845.09±4.00c	869.04±2.47a	839.97±3.00c	859.48±3.67b	0.000	0.199	0.685
SGR/% d-1	5.46±0.01c	5.49±0.00a	5.46±0.01c	5.48±0.01b	0.000	0.152	0.926
FCR	1.03±0.01b	1.01±0.01c	1.05±0.01a	1.04±0.01ab	0.000	0.000	0.839
FI/% d-1	5.55±0.01b	5.45±0.01c	5.62±0.00a	5.64±0.03a	0.000	0.000	0.255

AMP, antimicrobial peptide; SR, survival rate; IBW, initial body weight; FBW, final body weight; SGR, specific growth rate; WGR, weight gain rate; FCR, feed conversion ratio; FI, feed intake. Values are shown as means ± SEM (n = 6). Different letters in the same line represent significant differences (*P* < 0.05).

### 3.2 Whole-body composition and physical indexes

As the supplementation of AMP in diets increased, the crude protein of fish initially rose and then decreased, which was remarkably higher in the AMP-2 group than in other groups (*P* < 0.05). In the meantime, the supplementation of AMP in diets significantly reduced crude ash of fish (*P* < 0.05), while the crude lipid exhibited no significant difference (*P* > 0.05) (**Table 5**).

**Table 5:** The effects of dietary antimicrobial peptides piscidin 4 (TP4) on body composition of juvenile Nile Tilapia for 60-day feeding trial.

Parameters	AMP supplementation level				Polynomial contrasts		
	Control	AMP-2	AMP-4	AMP-8	*P*_-ANOVA_	*P*_-Linear_	*P*_-Quadratic_
Moisture/%	71.55±1.29	71.29±0.67	71.54±1.30	71.89±0.41	0.980	0.751	0.822
Crude protein/%	15.85±0.99^c^	17.21±0.33^a^	16.08±0.44^b^	16.42±0.13^b^	0.044	0.851	0.534
Crude lipid/%	3.91±0.28	4.71±0.48	4.65±0.45	5.15±0.33	0.051	0.115	0.297
Crude ash/%	6.13±0.30^a^	5.07±0.18^b^	5.10±0.22^b^	5.08±0.15^b^	0.005	0.010	0.015

AMP, antimicrobial peptide. Values are shown as means ± SEM (n = 6). Different letters in the same line represent significant differences (*P* < 0.05).

With the supplementation of AMP in diets increasing to 4.0 g/kg, there was no significant difference in CF (*P* > 0.05), while CF was significantly higher in the AMP-2 group than in the AMP-8 group (*P* < 0.05). HSI in the AMP-8 group was significantly higher than in other groups (*P* < 0.05), whereas there were no significant differences in VSI among groups (*P* > 0.05) (**Table 6**).

**Table 6:** The effects of dietary antimicrobial peptides piscidin 4 (TP4) on physical indexes of juvenile Nile Tilapia for 60-day feeding trial.

Parameters	AMP supplementation level				Polynomial contrasts		
	Control	AMP-2	AMP-4	AMP-8	*P*_-ANOVA_	*P*_-Linear_	*P*_-Quadratic_
VSI/%	9.01±0.35	9.59±0.28	8.39±0.43	8.95±0.89	0.495	0.656	0.734
HSI/%	1.04±0.10^b^	1.09±0.09^b^	1.07±0.09^b^	1.24±0.17^a^	0.019	0.029	0.449
CF	3.98±0.07^ab^	4.16±0.08^a^	4.06±0.14^ab^	3.69±0.20^b^	0.037	0.059	0.091

AMP, antimicrobial peptide; CF, condition factor; HSI, hepatosomatic index; VSI, viscerosomatic index. Values are shown as means ± SEM (n = 6). Different letters in the same line represent significant differences (*P* < 0.05).

### 3.3 Intestinal digestive enzymes

The supplementation of AMP in diets had no notable effects on the activity of intestinal AMS (*P* > 0.05). However, activities of intestinal LPS and TRY increased first and then decreased with the increase of AMP in diets (*P* < 0.05). Meanwhile, TRY in the AMP-2 group and LPS in AMP-2 and AMP-4 groups were significantly higher than in other treatments (*P* < 0.05) (**Table 7**).

**Table 7:** The effects of dietary antimicrobial peptides piscidin 4 (TP4) on intestine digestive enzymes of juvenile Nile Tilapia for 60-day feeding trial.

Parameters	AMP supplementation level				Polynomial contrasts		
	Control	AMP-2	AMP-4	AMP-8	*P*_-ANOVA_	*P*_-Linear_	*P*_-Quadratic_
TRY (U/mg prot)	0.42±0.01b	0.76±0.01a	0.42±0.02^b^	0.42±0.01^b^	0.013	0.769	0.043
LPS (U/g prot)	0.99±0.04b	1.21±0.05a	1.17±0.03a	0.93±0.04b	0.015	0.280	0.020
AMS (U/mg prot)	0.44±0.03	0.45±0.02	0.42±0.02	0.45±0.03	0.421	0.910	0.254

AMP, antimicrobial peptide; LPS, lipase; TRY, trypsin; AMS, α-amylase. Values are shown as means ± SEM (n = 6). Different letters in the same line represent significant differences (*P* < 0.05).

### 3.4 Hepatic antioxidant capacity

**Figure 1:**
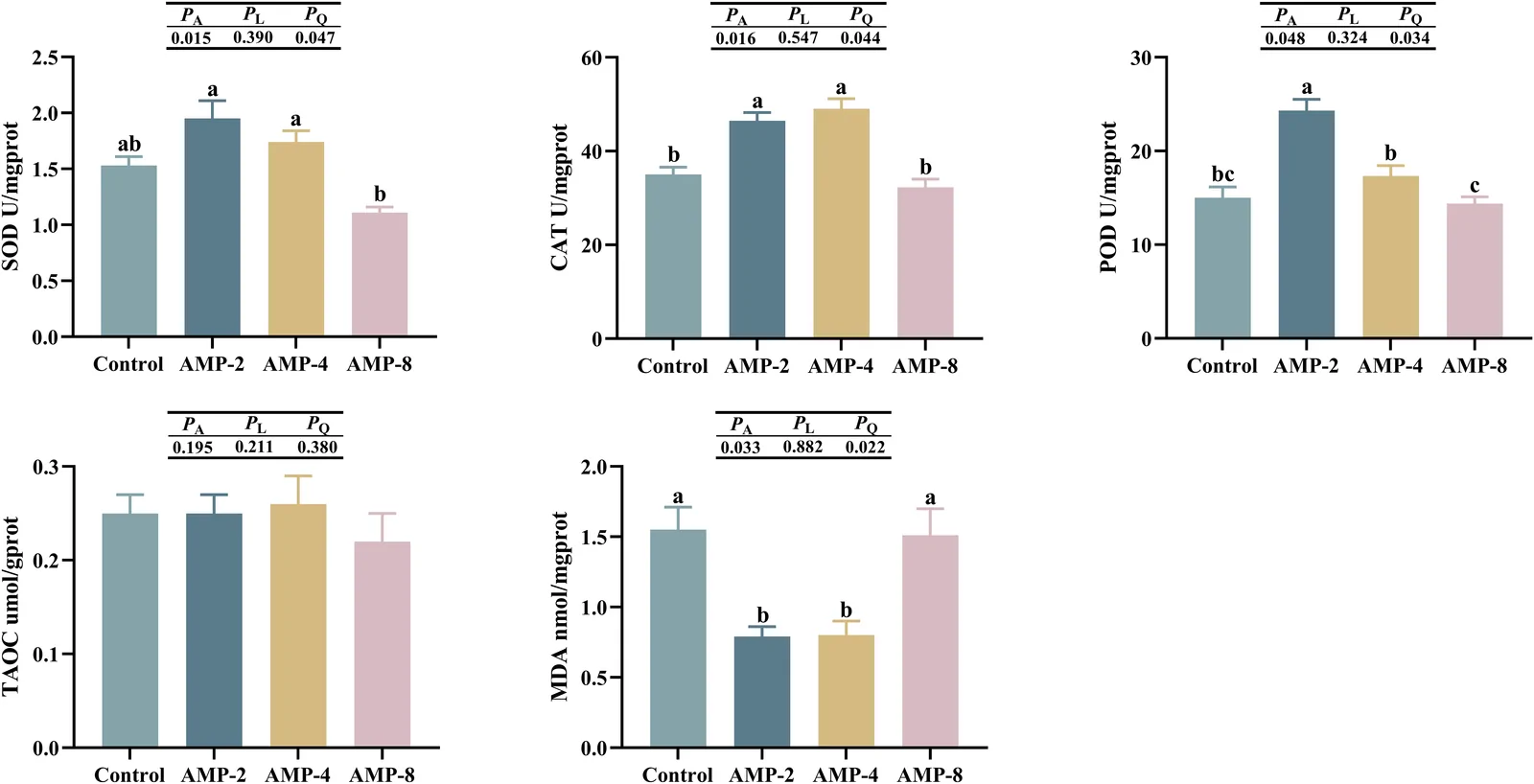
The effects of dietary antimicrobial peptides piscidin 4 (TP4) on hepatic antioxidant capacity of juvenile Nile Tilapia. Abbreviations: TAOC, total antioxidant capacity; SOD, superoxide dismutase; CAT, catalase; POD, peroxidase; MDA, malondialdehyde. Data are given as means ± SEM. One-way ANOVA was used and different letters above the columns indicate significant difference (*P* < 0.05).

As the supplementation of AMP in diets increased, activities of CAT and POD in liver first increased and then decreased (*P* < 0.05), whilst MDA content first decreased and then increased (*P* < 0.05). The activities of SOD in AMP-2 and AMP-4 were significantly higher than in AMP-8 (*P* < 0.05). In contrast to the control group, the AMP-2 group dramatically elevated activities of POD, and AMP-2 and AMP-4 groups elevated activities of CAT and reduced the content of MDA (*P* < 0.05). However, there were no significant differences observed in TAOC among four groups (*P* > 0.05) (**Figure 1**).

### 3.5 Hepatic immune responses

**Figure 2:**
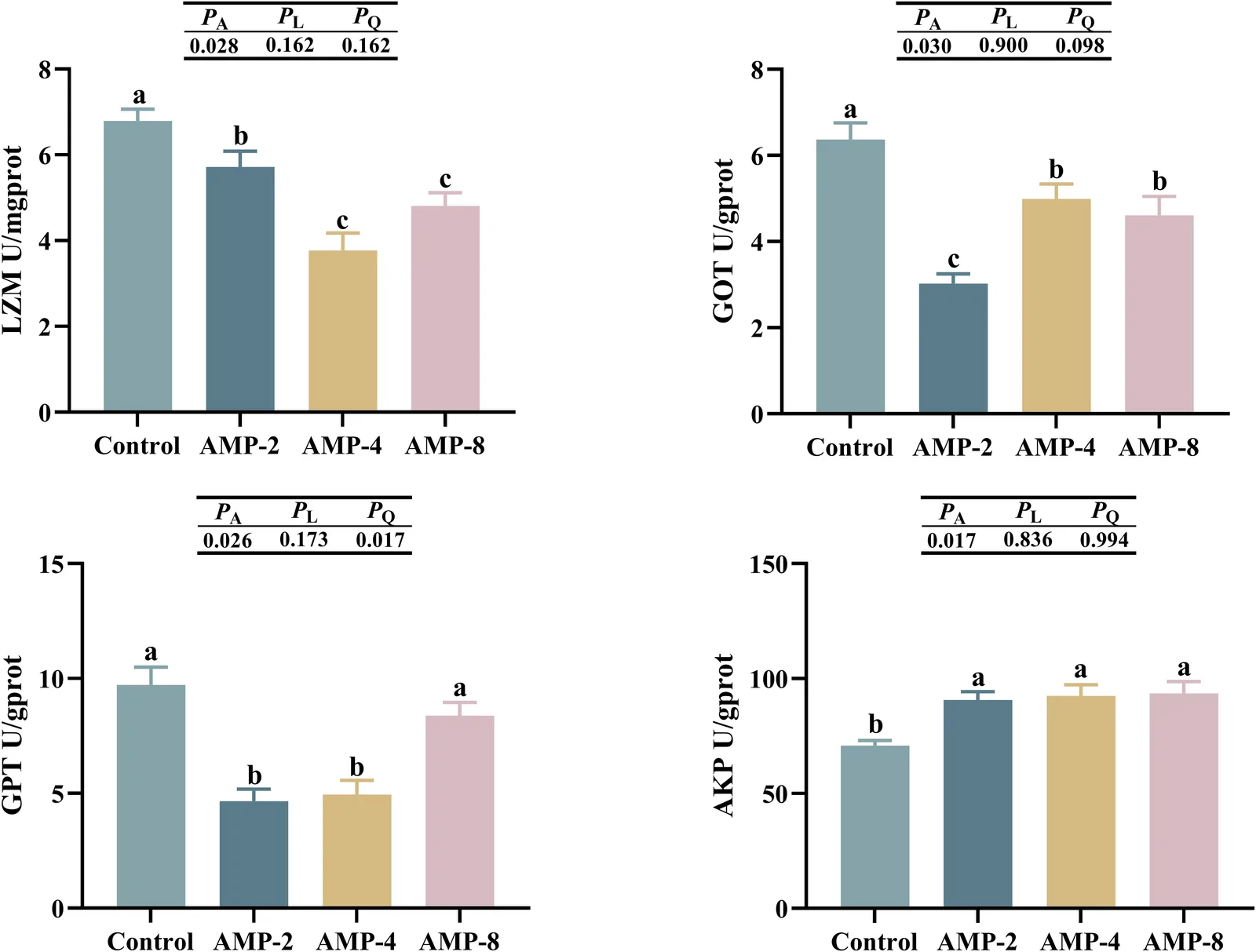
The effects of dietary antimicrobial peptides piscidin 4 (TP4) on hepatic immune responses of juvenile Nile Tilapia. Abbreviations: LZM, lysozyme; GOT, glutamic-oxalacetic transaminase; GPT, glutamic-pyruvic transaminase; AKP, alkaline phosphatase. Data are given as means ± SEM. One-way ANOVA was used and different letters above the columns indicate significant difference (*P* < 0.05).

In contrast to the control group, three groups supplemented AMP significantly reduced the activity of GOT, and elevated the activity of AKP (*P* < 0.05). As the supplementation of AMP in diets rose to AMP-4, the activity of LZM presented a downward trend (*P* < 0.05). However, as AMP levels increased, the activity of GPT showed a decline followed by an increase, and was markedly lower in AMP-2 and AMP-4 groups than in other groups (*P* < 0.05) (**Figure 2**).

### 3.6 Hepatic antioxidant-related gene expressions

**Figure 3:**
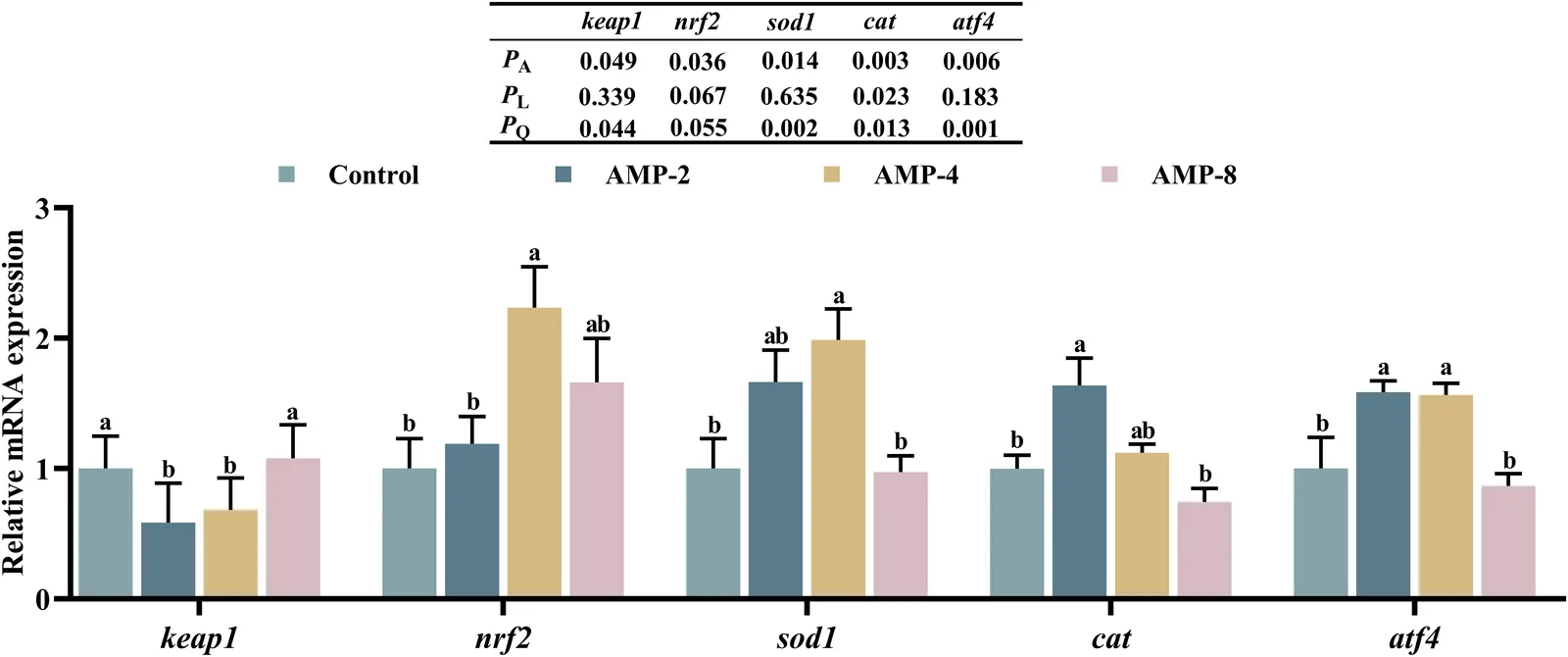
The effects of dietary antimicrobial peptides piscidin 4 (TP4) on hepatic antioxidant-related genes of juvenile Nile Tilapia. Abbreviations: *keap1*, kelch-like ECH-associated protein 1; *sod1*, superoxide dismutase 1; *cat*, catalase; *nrf2*, nuclear factor E2-related factor; *atf4*, activating transcription factor 4. Data are given as means ± SEM. One-way ANOVA was used and different letters above the columns indicate significant difference (*P* < 0.05).

With the levels of dietary AMP increasing, gene expression of hepatic *atf4* first rose and then fell, whereas *keap1* was the opposite (*P* < 0.05). Compared to the control group, AMP-2 and AMP-4 groups significantly upregulated the expression of *atf4* and downregulated the expression of *keap1*, while the AMP-2 group significantly upregulated the expression of *cat* and the AMP-4 group upregulated the expressions of *nrf2* and *sod1* (*P* < 0.05). Meanwhile, there was no significant difference in genes expression above between the control and AMP-8 group (*P* > 0.05) (**Figure 3**).

### 3.7 Hepatic immune-related gene expressions

**Figure 4:**
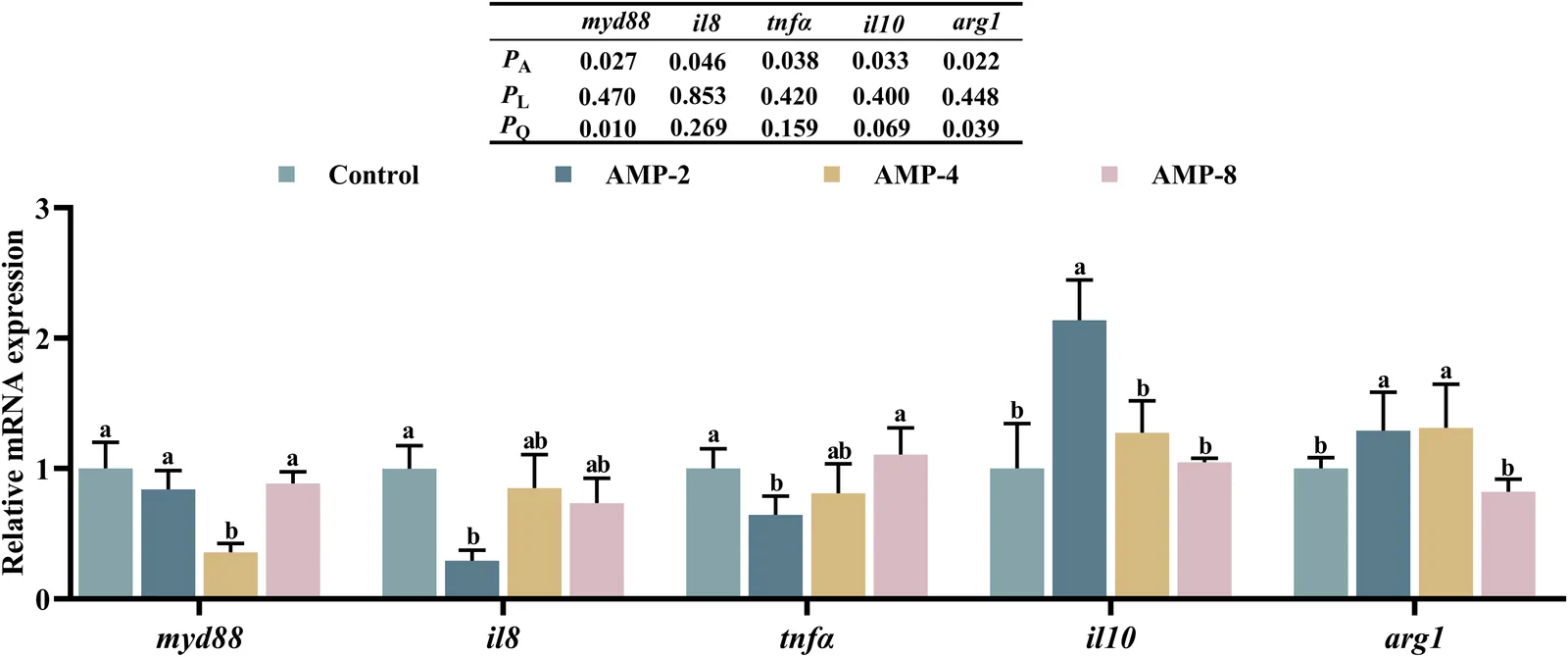
The effects of dietary antimicrobial peptides piscidin 4 (TP4) on hepatic immune-related genes of juvenile Nile Tilapia. Abbreviations: *myd88*, myeloid differentiation factor 88; *il8*, interleukin 8; *tnfα*, tumor necrosis factor α; *il10*, interleukin 10; *arg1*, arginase 1. Data are given as means ± SEM. One-way ANOVA was used and different letters above the columns indicate significant difference (*P* < 0.05).

As the supplementation of AMP in diets elevated, pro-inflammation-related gene *tnfα* expression first declined and then rose, while anti-inflammation-related gene *arg1* were the opposite (*P* < 0.05). Compared to the control group, AMP-2 and AMP-4 groups significantly upregulated the expression of *arg1*, whilst the AMP-2 group significantly downregulated the expressions of *il8* as well as *tnfα* and simultaneously upregulated the expression of *il10*, and the AMP-4 group markedly downregulated the expression of *myd88* (*P* < 0.05). Besides, no significant difference was observed in genes expression above between the control and AMP-8 group (*P* > 0.05) (**Figure 4**).

### 3.8 Post-challenge relative percent survival

**Figure 5:**
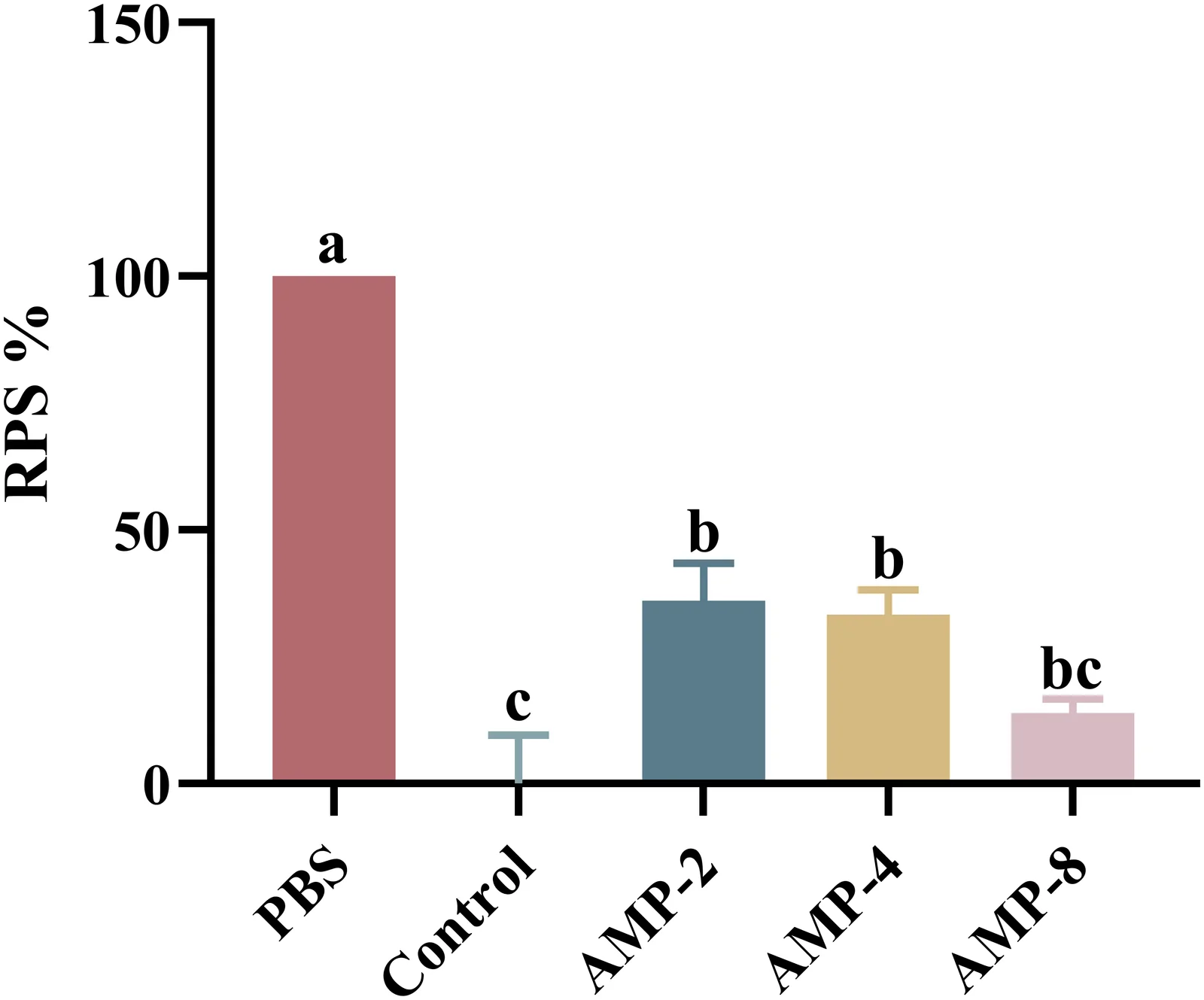
The effects of dietary antimicrobial peptides piscidin 4 (TP4) on RPS of juvenile Nile Tilapia after challenged with *Streptococcus agalactiae*. Abbreviations: RPS, relative percent survival; PBS, phosphate buffered saline. Data are given as means ± SEM. One-way ANOVA was used and different letters above the columns indicate significant difference (*P* < 0.05).

The supplementation of AMP in diets improved the survival rate of tilapia after challenged with *S. agalactiae*. Compared with the control group, AMP-2 and AMP-4 groups significantly elevated RPS of fish under *S. agalactiae* (*P* < 0.05) (**Figure 5**).

## 4 Discussion

In the present study, endogenous tilapia antimicrobial peptide TP4, which is a host-derived cationic peptide with broad-spectrum bacteriostatic activity against aquatic pathogenic bacteria (Ting et al., 2018; Liu et al., 2021), was incorporated into extruded compound feeds as a functional additive. The TP4 content in experimental diets was measured, showing consistency with the formulated values (recovery rate 96.5%-97.3%). The WGR and SGR of juvenile tilapia were optimized and then suppressed as dietary AMP supplementation increased. A dietary AMP of 2.0 g/kg appeared to be appropriate according to SGR. The results indicated that the diet supplemented with appropriate levels of AMP could improve growth rate, which was consistent with findings in red drum (*Sciaenops ocellatus*) and large yellow croaker (Zheng et al., 2016; Ge et al., 2019; An et al., 2023). Meanwhile, the present study suggested that the crude protein content and CF of fish were all markedly increased, the crude lipid was not significantly affected, and the crude ash was significantly reduced, indirectly indicating that the enhanced growth performance of tilapia fed the AMP-2 diet might be allocated to whole-body protein deposition. Future studies should detect muscle protein turnover-related gene markers such as *mtor* and *murf1*, to further validate the regulatory effects of TP4 on protein synthesis and degradation. Nevertheless, conflicting outcomes regarding dietary AMP supplementation in terms of growth performance were also documented in the previous studies. For example, no growth-enhancing responses were observed in orange-spotted grouper, Nile Tilapia and African cichlid (*Sciaenochromis fryeri*) (Yokoyama et al., 2006; Welker et al., 2007; Moradian et al., 2018). Variations in growth performance across different studies may be attributed to distinct physiological functions of AMPs from different sources, as well as differential metabolic responses of various fish species to these peptides (Cunha et al., 2020; Wang et al., 2023). In fish, an increased HSI is usually not caused by hepatocyte proliferation, but by massive lipid droplet accumulation within hepatocytes, leading to hepatocyte vacuolization and liver enlargement (Xie et al., 2020). Compared with the control group, the AMP-8 group showed an increased growth rate, whereas HSI was significantly elevated, implying that high-dose AMP may, to some extent, affect weight gain by influencing visceral lipid weight.

This study also found that the AMP-2 group improved the feed utilization of tilapia, as evidenced by the significantly reduced FCR. Following feed ingestion, digestion and absorption mainly occur in the intestine, and these processes rely heavily on the function of intestinal digestive enzymes (Gisbert et al., 2009; Zhang et al., 2022a; Berge et al., 2023). In the present study, the diet supplemented with 2g/kg AMP significantly elevated the activities of intestinal TRY and LYS in tilapia, in line with previous reports on tilapia and marbled eel (*Anguilla marmaorata*) (Shi et al., 2014; Zhai et al., 2016). In general, higher digestive enzyme activities facilitate greater feed digestibility and utilization efficiency (Zhao et al., 2022), which partially explains the reduction in FCR in fish fed the AMP-2 diet. Notably, FI was reduced in tilapia fed the AMP-2 diet in this study. This phenomenon may be attributed to the higher growth rate and larger body size of fish in the AMP-2 group, as larger-sized fish generally exhibit a lower FI compared to the smaller ones (Yuan et al., 2018; Licitra et al., 2024).

Changes in growth metabolism are usually accompanied by synchronous variations in the antioxidant status of the organism (Li et al., 2021; Fang et al., 2022). To clarify the regulatory effects of AMPs on the antioxidant capacity of tilapia, a set of key antioxidant biochemical indicators were measured in the present study. The AMP-2 group significantly elevated the antioxidant enzymes (CAT, SOD and POD) and reduced the MDA level in the liver of tilapia. CAT, SOD and POD are the main antioxidant enzymes and antioxidants, playing an important role in removing reactive oxygen species (Cheng et al., 2018). MDA can indirectly reflect fish cell damage and the severity of oxidative stress (Fei et al., 2022). Therefore, combined with the changes in these indicators, the diet supplemented suitable AMP would improve the antioxidant property of tilapia, which was in keeping with the conclusions of previous studies, such as orange-spotted grouper, largemouth bass and crucian carp (*Carassius auratus*) (Su et al., 2019; Li et al., 2020; Dong et al., 2025). Consistent with the above results, this study further found that diets AMP-2 or AMP-4 groups downregulated *keap1* gene and upregulated *nrf2*, *sod1*, *cat* and *atf4* genes in the liver. The down-regulated *keap1* releases *nrf2* for nuclear translocation, which further transcriptionally activates downstream antioxidant genes including *sod1* and *cat* (Bollong et al., 2018; Huang et al., 2022). Consistently, the elevated mRNA abundances correspond to higher hepatic CAT and SOD enzyme activities, which accelerate reactive oxygen species elimination and consequently reduce MDA content, the key marker of lipid peroxidation and oxidative injury (Karna et al., 2019). Such transcriptional-biochemical coupling may underpin the improved hepatic antioxidant capacity observed in AMP-2 and AMP-4 groups. Simultaneously, as a master regulatory factor of the integrated stress response, the upregulated expression of *atf4* can alleviate excessive cellular oxidative damage and maintain systemic antioxidant homeostasis (Rached et al., 2010). Interestingly, TAOC was found to remain unaffected. TAOC is jointly determined by enzymatic and non-enzymatic antioxidants (Lubiński et al., 2021; Xiao et al., 2021). Although feeding AMP-supplemented diets elevated the activities of antioxidant enzymes in this study, the organism may regulate the metabolism of partial non-enzymatic substances via a compensatory effect to sustain overall antioxidant homeostasis (Díaz-De la Cruz et al., 2021).

Improved antioxidant homeostasis can synergistically potentiate immune responses in fish (Cruz-Chamorro et al., 2020; Kim et al., 2021), and antimicrobial peptides inherently possess both bacteriostatic activity and immunomodulatory potential (Zhang et al., 2014). Subsequently, the study found that AMP-2 and AMP-4 groups markedly reduced GOT and GPT and elevated AKP activities in the liver. GOT and GPT are marker enzymes of hepatocyte injury, the decrease of which indicated that AMPs alleviated hepatic oxidative and inflammatory damage and preserved the integrity of hepatocyte membrane (Saat et al., 2016). Likewise, as an enzyme involved in immune defense and metabolism, the elevated hepatic AKP activity suggested enhanced hepatic immune and metabolic functions in tilapia (Yin et al., 2022). The core of immune homeostasis lies in the dynamic balance of pro-inflammatory and anti-inflammatory signals (Li et al., 2016; Findeisen et al., 2022).

Further molecular results showed that dietary supplementation with AMPs may inhibit the gene expression of key molecules in pro-inflammatory pathways and pro-inflammatory factors, while upregulating the expression of anti-inflammatory related genes. The differential expression profiles of inflammatory-related genes further illustrate the immunomodulatory mechanism of dietary TP4. *Myd88* acts as a central adaptor in the TLR-mediated pro-inflammatory cascade, and its overexpression initiates the transcription of pro-inflammatory mediators *il8* and *tnfα*, which aggravate hepatic inflammatory lesions upon pathogenic stimulation (Karmakar and Mandal, 2021). The 2 and 4 g/kg AMP groups markedly suppressed the transcription of *myd88*, *il8* and *tnfα*, indicating that moderate TP4 may block excessive activation of the pro-inflammatory signaling pathway. Meanwhile, anti-inflammatory genes *il10* and *arg1* were significantly upregulated in these two groups. Furthermore, *il10* suppresses the synthesis of pro-inflammatory cytokines (Röring et al., 2025), and *arg1* facilitates M2 macrophage polarization to alleviate inflammatory damage (Liu and Liang, 2026). The coordinated downregulation of pro-inflammatory genes and upregulation of anti-inflammatory genes demonstrates that appropriate TP4 addition would maintain inflammatory homeostasis in tilapia liver. This molecular regulatory pattern directly accounts for the decreased hepatic injury enzymes, including GOT and GPT (Huang et al., 2015), observed in AMP-2 and AMP-4 groups, supporting the immunoprotective effect of tilapia-derived AMP. Furthermore, the results of the *S. agalactiae* challenge test directly confirmed that diets supplemented with suitable AMP would exert positive effects on enhancing disease resistance in tilapia, agrees with prior studies on abalone (*Haliotis discus hannai*) and Pengze crucian carp (*Carassius auratus* var. *Pengze*) (Chen et al., 2020; Wang et al., 2021a). However, dietary supplementation with AMP at 8 g/kg failed to improve the antioxidant and immune indicators of tilapia, indicating that excessive AMP may not further enhance the antioxidant and immune performance of tilapia. This observation may be attributed to disturbed physiological homeostasis, excessive peptide degradation or saturated target receptors under high peptide dosage, suggesting that antimicrobial peptides may have an optimal dose window in aquaculture, and higher supplementation does not guarantee better biological efficacy (Zhang et al., 2025). Taken together, appropriate AMP in diets may protect liver tissues and optimize immune status by regulating the balance of inflammatory signals, thereby improving disease resistance of tilapia.

Published commercial exogenous AMPs generally improve aquatic growth and antioxidant capacity but suffer poor heat stability, demanding low-temperature post-coating to avoid activity loss (Bai et al., 2024). By contrast, endogenous TP4 would possesse thermostable amphipathic helical conformation supported by abundant cationic arginine residues (Zhou et al., 2025), might retaining activity after briefly extrusion and 55°C drying (Tai et al., 2021). Functionally, TP4 may synchronously upregulates *nrf2* and suppresses *myd88/tnfα*, delivering balanced anti-oxidative and anti-inflammatory effects. However, TP4 has limitations in terms of cost and application scenarios and cannot totally replace antibiotics to date.

Some limitations of the present study should be noted. First, due to constraints in the experimental methodology, only the content of TP4 in diets was measured, which can only provide indirect evidence for TP4 activity. Second, this study focus on single fish developmental stage and lacks intestinal microbiota profiling and *in-vitro* cellular validation about direct molecular mechanism. Third, only artificial acute pathogen challenge was performed, and multi-stress co-exposure testing was lacking. Final, given that TP4 material costs lack industrial-scale optimization, no cost-benefit analysis was conducted here. These shortcomings warrant further investigation in future studies.

## 5 Conclusion

In summary, diets supplemented with 2–4 g/kg AMP piscidin 4 (TP4) improve feed utilization and enhance growth rate of Nile Tilapia. Meanwhile, diets with 2–4 g/kg TP4 would strengthen antioxidant capacity by regulating the expression of antioxidant-related genes in liver and elevate immune function and disease resistance via modulating hepatic pro- and anti-inflammatory gene expression in liver to balance inflammatory signaling cascades, consequently maintaining fish health. Taken together, TP4 at 2-4 g/kg appears to confer benefits to the growth and health of tilapia in the present study, while ≥8 g/kg TP4 may generate suboptimal physiological outcomes on tilapia. Appropriate doses of TP4 may exert synergistic effects on growth promotion and fish health, with potential mechanisms awaiting further investigation.

## Data Availability

The datasets presented in this study can be found in online repositories. The names of the repository/repositories and accession number(s) can be found below: https://www.ncbi.nlm.nih.gov/, KJ126772.1;XM_003447926.3;XM_019361816.2;XM_003447296.4;XM_003459356.5;100701098;100700534;100534479;100697746;100694754;100706482.
