# Supplementary material for: Effects of dietary antimicrobial peptides on growth performance, antioxidant capacity, immune response and disease resistance of juvenile Nile tilapia (Oreochromis niloticus)
Source: Front Physiol. 2026 Sep 11;17:1936199. doi: 10.3389/fphys.2026.1936199 (PMC13612361; doi:10.3389/fphys.2026.1936199)
Supplement: Supplementary file 1 [file Table1.docx]

**Table S1**

Mortality of Nile tilapia infected with different concentrations of *Streptococcus agalactiae* in LD_50_ pre-experiment.

| Bacterial concentration (CFU/mL) | Injection dosage per fish (CFU) | Cumulative mortality (7 d, %) |
| --- | --- | --- |
| 1.0×10⁶ | 1.0×10⁵ | 11.67 |
| 5.0×10⁶ | 5.0×10⁵ | 28.33 |
| 1.0×10⁷ | 1.0×10⁶ | 46.67 |
| 5.0×10⁷ | 5.0×10⁶ | 75.00 |
| 1.0×10⁸ | 1.0×10⁷ | 93.33 |
| Control (PBS) | 0 | 0.00 |

Note: **Pre-experiment for the determination of median lethal concentration (LD_50_).** Prior to the formal bacterial challenge test, a pre-experiment was conducted to determine the median lethal concentration (LD_50_) of *Streptococcus agalactiae* in juvenile Nile tilapia, so as to screen the optimal bacterial concentration for the normal challenge assay. The preserved *S. agalactiae* strain was activated and cultured following the bacterial suspension preparation method described in the manuscript. After gradient dilution with sterile PBS, five different bacterial concentrations were set for the pre-experiment. Healthy tilapia with uniform body size were randomly divided into five bacterial infection groups and one blank control group, with 20 fish per replicate and three biological replicates per group. Fish in each infection group were intraperitoneally injected with 0.1 mL of bacterial suspension at corresponding concentrations, while the control group was injected with an equal volume of sterile PBS. The mortality of tilapia in each group was continuously recorded within 7 days after infection, and the LD_50_ value of *S. agalactiae* against tilapia was calculated via the improved Karber method. Combined with the pre-experimental results, 1.0×10⁷ CFU/mL was selected as the final bacterial concentration for the formal challenge test, which could cause moderate and stable mortality in tilapia without excessive death or invalid infection.

**Table S2**

The expected amplicon size for each primer pair in this study.

| Genes | Gene bank No. | Amplicon size (bp) |
| --- | --- | --- |
| *β-actin* | KJ126772.1 | 121 |
| *keap1* | XM_003447926.3 | 142 |
| *sod1* | [100701098](https://www.ncbi.nlm.nih.gov/gene/100701098/) | 108 |
| *cat* | XM_019361816.2 | 136 |
| *nrf2* | XM_003447296.4 | 154 |
| *atf4* | XM_003459356.5 | 117 |
| *myd88* | 100700534 | 163 |
| *il8* | [100534479](https://www.ncbi.nlm.nih.gov/gene/100534479/) | 98 |
| *tnfα* | [100697746](https://www.ncbi.nlm.nih.gov/gene/100697746/) | 131 |
| *il10* | [100694754](https://www.ncbi.nlm.nih.gov/gene/100694754/) | 148 |
| *arg1* | [100706482](https://www.ncbi.nlm.nih.gov/gene/100706482/) | 182 |

**Table S3**

TP4 contents of diets detected in this study.

| Diets | AMP supplementation (g/kg) | Detected AMP (g/kg) | Recovery rate (%) |
| --- | --- | --- | --- |
| Control | 0 | 0.01 | - |
| AMP-2 | 2.0 | 1.93±0.06 | 96.5% |
| AMP-4 | 4.0 | 3.89±0.09 | 97.3% |
| AMP-8 | 8.0 | 7.78±0.14 | 97.3% |

Note: **The brief description of detected method of dietary antimicrobial peptides (AMPs).** The actual AMP content in diets was determined by reversed-phase high-performance liquid chromatography (RP-HPLC). Briefly, 5.0 g of ground feed sample was extracted with 25 mL of 0.1% (v/v) trifluoroacetic acid (TFA) in acetonitrile/water (50:50, v/v) under sonication for 30 min at room temperature. The supernatant was collected after centrifugation at 10,000 × *g* for 15 min at 4 °C, and the residue was re-extracted once. The combined supernatants were filtered through a 0.22 μm membrane and analyzed on an HPLC system equipped with a C18 column (4.6 × 250 mm, 5 μm) using a linear gradient of acetonitrile in 0.1% TFA-water at a flow rate of 1.0 mL/min, with detection wavelength set at 220 nm. AMP concentration was quantified against a standard curve generated using purified AMP standard. All samples were analyzed in triplicate.

**Table S4**

TP4 contents of diets after the feeding trial detected.

| Diets | AMP supplementation (g/kg) | Detected AMP (g/kg) | Recovery rate (%) |
| --- | --- | --- | --- |
| Control | 0 | - | - |
| AMP-2 | 2.0 | 1.80±0.07 | 90.0% |
| AMP-4 | 4.0 | 3.58±0.10 | 89.5% |
| AMP-8 | 8.0 | 7.23±0.10 | 90.4% |
